# PP104 Utility Of Chemiluminescence Detection Of Anti-Domain 1 β2 Glycoprotein I Antibodies In Antiphospholipid Syndrome

**DOI:** 10.1017/S0266462324002654

**Published:** 2025-01-07

**Authors:** Tasmania del Pino-Sedeño, Aránzazu Hernández-Yumar, Cristina Valcárcel-Nazco, Aythami De Armas-Castellano, Yadira González-Hernández, Diego Infante-Ventura, Juan Ignacio Martín Sánchez, Mar Trujillo-Martín

## Abstract

**Introduction:**

Patients with antiphospholipid syndrome (APS) present a significant risk of thrombotic events or morbidity during pregnancy, associated with the presence of persistently positive antiphospholipid antibodies (aPL). The risk stratification is crucial to adopt primary prophylaxis measures. With this objective, assays for the in vitro detection of anti-domain 1 β2 glycoprotein I (aD1 IgG) antibodies by chemiluminescence immunoassay (CLIA) have been developed.

**Methods:**

We conducted a systematic review with meta-analyses on the effectiveness of incorporating aD1 IgG detection by CLIA for the identification of patients with APS at high risk of thrombosis or morbidity during pregnancy. A cost analysis was also conducted using a decision tree model from the perspective of the Spanish National Health System and a time horizon of three months. We ran extensive sensitivity analyses. Twelve diagnostic performance studies with a total of 3,570 patients were selected.

**Results:**

The sensitivity and specificity of aD1 IgG added to the conventional aPL criteria for obstetric and thrombotic manifestations ranged from 15 to 16 percent, and from 94 to 97 percent, respectively. The sensitivity of the detection of aD1 IgG in isolation for thrombotic and obstetric manifestations was 29 and 48 percent, and specificity was 60 percent, respectively. The GRADE quality of evidence was very low due to the risk of bias and indirectness issues. The incorporation of the aD1 IgG detection involves a higher cost per patient than the usual clinical practice, with an incremental difference of EUR24.04 (USD26.01).

**Conclusions:**

Despite its potential, the incorporation of the aD1 IgG detection by CLIA as an additional test to the determination of classical aPL for the identification of patients with APS at high thrombotic or obstetric risk may not be feasible due to the very low available evidence on diagnostic performance and increased costs.

